# Exploring User Experiences of an Augmented Reality Smartphone App Prescribing Exercise for Children and Young People With Cancer: Results From a Qualitative Study

**DOI:** 10.2196/76855

**Published:** 2026-04-22

**Authors:** Hayley Marriott, Kim Straun, Alba Solera-Sanchez, Eila Watson, Stanley Windsor, Marie A Neu, Elias Dreismickenbecker, Joerg Faber, Peter Wright

**Affiliations:** 1 School of Sport, Nutrition and Allied Health Professions, Oxford Brookes University, Headington Road, Oxford, OX3 0BP, United Kingdom, +44 01865484260; 2 School for Policy Studies, University of Bristol, Bristol, United Kingdom; 3 Oxford Institute of Applied Health Research, Oxford Brookes University, Oxford, United Kingdom; 4 Childhood Cancer Center Mainz, University Medical Center of the Johannes Gutenberg-University Mainz, Mainz, Germany

**Keywords:** mHealth, AR, qualitative research, physical activity, exercise, childhood cancer, mobile health, augmented reality, mobile phone

## Abstract

**Background:**

Mobile health (mHealth), and specifically smartphone apps, have grown exponentially in both functionality and accessibility and are becoming an important component of health care. Research exploring the use of mHealth for managing or treating chronic diseases, such as cancer, has shown promising effects. Yet, comparatively little work has examined how such technologies can enhance exercise interventions for young people with cancer. To optimize the effectiveness of mHealth in these contexts, it is essential to build a stronger evidence base on user experience.

**Objective:**

This study aimed to investigate how healthy children and young people engaged with an augmented reality (AR) app developed specifically for children and young people undergoing cancer treatment, and to identify design features that may support engagement and behavior change in the intended clinical population.

**Methods:**

School and university students, aged 8‐21 years, were eligible to participate in the study. Practical workshops allowed participants to engage with the AR exercise app before taking part in focus groups to explore user experiences. Data were analyzed using qualitative content analysis, which also involved a critical friend approach using 2 researchers (HM and KS). Suggested improvements were mapped against the motivational affordances’ taxonomy.

**Results:**

A total of 39 participants aged 8‐21 years took part in the focus group study. Participants found the demonstrations and varied exercises useful but expressed some concerns regarding data safety and functionality of the novel AR avatar. It was proposed that additional educational components, challenges, and rewards, as well as a customizable avatar, social support features, and audio instructions for a more inclusive design would be desirable and could enhance user experience. When mapped against the motivational affordances taxonomy, the suggested improvements aligned primarily with mechanisms of user education, challenges, feedback, cooperation, and comparison.

**Conclusions:**

This study provides an understanding of how apps that prescribe exercise can be optimized to enhance motivation and user experience. By assessing feedback and suggestions for improvements, the findings highlight key design features that may support engagement. While this initial work focused on healthy, age-matched participants, further evidence specifically in children and young people with a childhood cancer diagnosis is needed.

## Introduction

Over the past 50 years, chronic health conditions among children and young people have risen on a global scale [1]. Conditions such as cancer, type 2 diabetes, obesity, and mental health disorders are increasingly prevalent in this population, resulting in a substantial burden on health care systems [2]. Addressing this issue requires a comprehensive, multidisciplinary approach, incorporating preventive public health measures, early and targeted interventions, and health care systems that cater to the physical and psychological dimensions of chronic illnesses in children and young people [3]. The use of technologies within health care may offer an important role in the management of long-term conditions in pediatric populations [4].

Specific research in patients with cancer has reported that digital interventions have elicited positive outcomes, including improving adherence to medication [5], symptom management [6], physical activity levels [7], and mental well-being [8]. Digital tools can empower young patients to take an active role in their health management [9], as well as having the potential to alleviate some of the pressures (eg, staff shortages, limited capacity of services and facilities, and increased demand) on health care systems by supporting disease management outside of traditional clinical settings.

Children and young people undergoing treatment for cancer have some unique barriers to physical activity. These include (1) treatment-related side effects, such as fatigue and pain; (2) psychological barriers, such as fear of injury and low mood; and (3) organizational barriers, such as limited clinical space and lack of sports equipment during home stays [10].

Addressing these barriers requires accessible, personalized solutions. Digital interventions have grown exponentially in both functionality and accessibility, emerging as an increasingly important component of health care delivery [11]. Interventional evidence suggests that using mobile health (mHealth) tools can benefit those managing chronic conditions, such as cancer, by offering a more personalized approach to real-time health management [12]. mHealth tools have been shown to provide a medium for personalized care, continuous monitoring, and patient engagement, therefore improving clinical outcomes and enhancing quality of life for patients [13,14]. mHealth has opened doors for the development of innovative smartphone apps tailored to meet the health needs of specific populations, such as pediatric patients with cancer [15].

More specifically, the integration of augmented reality (AR) in mHealth tools has gained traction in recent years [16]. AR technology overlays digital information on places or objects in the real world [17], as seen in apps, such as Pokémon Go (Niantic Inc). It is expected that incorporating AR within existing tools could enhance user satisfaction and facilitate long-term engagement by using more graphic-focused screen design and multimedia message presentation, as well as clear demonstrations and instructions by an AR avatar [18]. There is a previous systematic review [19] exploring the effectiveness of AR interventions for physical activity promotion and improving health outcomes. This evidence in children and young people reported that AR helped participants increase their physical activity levels as well as their engagement and motivation toward physical activity. Additionally, social interactions and psychological distress were also improved [19]. However, there is a lack of research on the user experience of AR apps specifically designed for promoting exercise participation during cancer rehabilitation.

Development and implementation of mHealth tools and specifically AR apps, in pediatric populations, especially for long-term, maintained engagement, presents significant challenges, emphasizing the need to use theoretical frameworks as well as evidence-based and user-centered approaches [20,21]. In recent years, there has been an increased acknowledgment of behavior change techniques, such as goal setting, motivational messaging, and outcome feedback, in the design of mHealth tools and programs [21]. Current guidance for designing and evaluating mHealth tools has specified that it is of particular importance that behavior change techniques are used as a basis for development to ensure content is theory-driven and follows evidence of best practice [22]. The app that was designed and evaluated as part of the research outlined here contains features based on behavior change techniques, such as feedback on behavior, instruction on how to perform the behavior, and demonstration of the behavior [23]. While these techniques are promising for integration in interventions promoting physical activity, there is limited evidence on their application in more complex mHealth tools, such as apps using AR. To address this gap, additional strategies, including motivational affordances, should be considered.

Motivational affordances are defined as design features, such as gamification and personalized feedback, that focus on increasing user engagement, distinguishing them from the internal cognitive processes emphasized in behavior change techniques [24,25]. The success of apps relies on user engagement, which is based on the user’s motivation. Satisfaction, efficiency, and engagement define the overall user experience; however, apps do not always provide the expected results and rarely investigate user engagement [26]. For this purpose, the integration of disciplines of usability and motivational psychology could be achieved through research in motivational affordances [27]. Motivational affordance mechanisms and design elements could be used to satisfy the user’s motivational requirements (ie, psychological, cognitive, social, and emotional); thus, there have been calls for motivational affordances to be applied in mHealth design. Theory-based approaches for the evaluation of apps are particularly useful due to their specific technology designs, which seek to tap into users’ motivations, so they are captivated by them and really seek to use them [28]. However, there is a particular lack of evidence evaluating the usefulness of and user preferences for motivational affordances in exercise prescription apps for children and young people with cancer [29,30]. It is vital to specifically assess the potential for integrating motivational affordances in exercise prescription apps, since their adequacy and specificity are key in populations with chronic diseases [31].

Due to the novel nature of this app, there is a need for extensive evaluation of the app’s functionality and usability, particularly regarding the navigation, layout, instructions, and AR demonstrations, before testing with children and young adults with cancer. The findings of this study provide valuable insights, which are more appropriately assessed in a more controlled setting, such as schools and healthy peers [32]. Participants of this study were age-matched to the target population for which the app was being developed.

Thus, the aim of this study was to explore the experiences of school pupils and university students of an AR smartphone app designed to prescribe exercise for children and young people with cancer. The study also aims to map participants’ suggestions for improvement onto motivational affordance mechanisms and design elements. Understanding user preferences allows developers to refine features, improve accessibility, and create more engaging and effective mHealth tools for exercise prescription [32].

## Methods

### Overview

The methods (including app design, recruitment, data collection, and data analysis) have been comprehensively described in a previous publication [32]. In reporting of methods, the 32-item COREQ (Consolidated Criteria for Reporting Qualitative Research) checklist has been used (Checklist 1) [33].

### AR App Development

The AR app was developed to support an individualized exercise program for children and young people undergoing cancer treatment, particularly when face-to-face provision was not possible, the child was at home, or they were immunocompromised. Its design was guided by an interdisciplinary team of software developers, digital health researchers, and pediatric oncology exercise professionals. Key features were implemented with the needs of children with cancer in mind, including professional input during onboarding to record diagnosis and contraindications, child-reported fatigue and activity levels, and a pre-session questionnaire to screen for new symptoms. The app offers upper body, lower body, and core exercises with seated and standing options, and a rate of perceived exertion scale that automatically adjusts exercise volume if intensity is inappropriate.

### Recruitment and Setting

Participants were recruited through schools and universities. Eligibility criteria are presented in Textbox 1.

Textbox 1. **Participant eligibility criteria.**

Children and young people in

- Years 4, 5, 6, 7, 8, and 9 of primary school and secondary school, aged 8-14 years old
- Sixth form in secondary school, aged 16-18 years old
- University, aged 19-21 years old

Proficient in the English language

- Able to take part in a session up to 30 min of low-moderate intensity body weight exercises

Participants were recruited through convenience sampling of age-matched children and young people, representing a mix of rural and urban schools, along with 1 university group. During the recruitment of primary school children, the research team liaised with the school and its teachers, who passed on information about the study to potential participants and legal guardians. Participants were told why the research was being conducted and that the app had been developed for children with cancer in mind. For secondary school pupils, members of the team visited the school to explain the purpose of the study and outline what involvement in the workshops and focus groups would entail. For university students, the team provided details about the study by attending a selection of lectures.

### Ethical Considerations

This research has received ethical approval from the Oxford Brookes University Faculty of Health and Life Sciences Research Ethics Committee (David E. Evans, Paul Hough, Robyn Curtis, and Jo Brett; approval 211547). Written informed consent and assent, including parental consent where applicable, were required to participate in the project in accordance with the project approvals granted by Oxford Brookes University Faculty of Health and Life Sciences Research Ethics Committee (registration 211547). All data were fully anonymized before analysis, with any identifying information removed to ensure participant confidentiality. Participation was voluntary, and individuals could withdraw at any time without penalty. Participants did not receive any compensation for their involvement.

Before the workshops, non–user-facing onboarding steps were completed to ensure that participants could access the main functions of the app, even though they did not have a cancer diagnosis. This setup involved selecting the following default options: all body areas were eligible for exercise, activities were not limited to seated movements, participants did not use a prosthetic limb, and they did not experience peripheral neuropathy. At the beginning of each workshop, participants were told why the research was being conducted and that the app had been developed for children with cancer in mind. None of the individuals invited to take part had used or interacted with the AR app.

**Figure 1.**
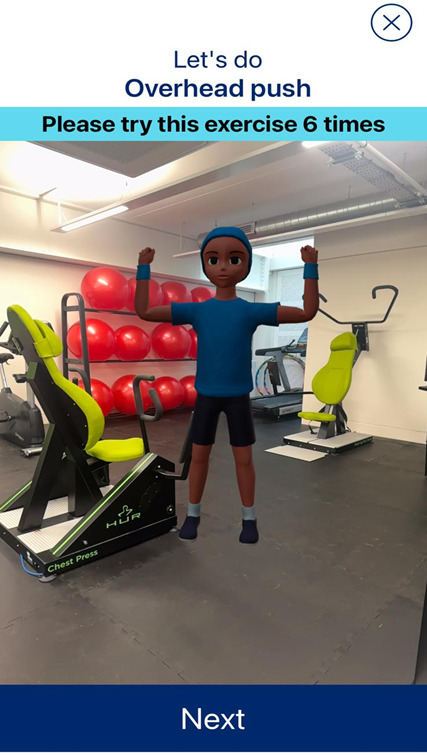
App screen during exercise.

**Figure 2.**
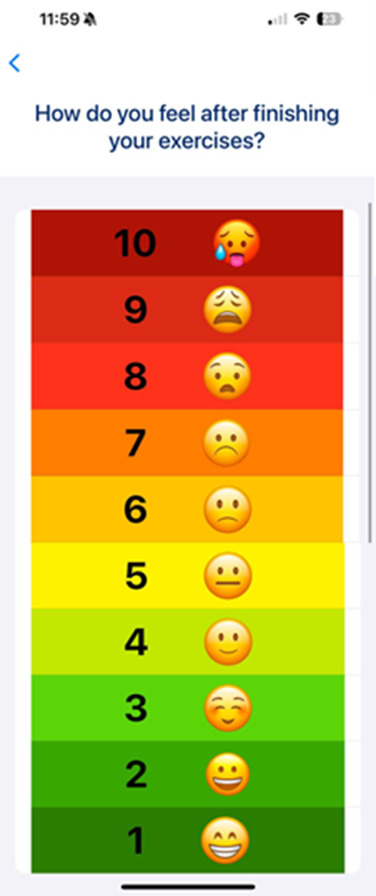
Rate of perceived exertion scale.

Participants were given a phone and then progressed through the user-facing onboarding sequence. Once this was completed, participants completed an individualized exercise session, following the AR avatar demonstrations (Figure 1). Each workout consisted of 6 exercises, with the number of repetitions (1-6) and sets (1-3) automatically adjusted by the app’s algorithm based on responses provided during the onboarding process. Following the session, participants completed a postsession questionnaire, which included a rating of perceived exertion measure (Figure 2).

Subsequently, participants took part in focus group discussions to explore the user experience of using the AR app and had the opportunity to share suggested improvements. For the focus groups, a semistructured interview guide was used (Table 1).

**Table 1.** Semistructured interview guide for focus groups.

Topic and guiding questions	Prompts
Smartphone app perceptions	
What are your thoughts on using a smartphone app to help you do exercise?	What kind of app are you thinking of? How have your thoughts changed from before the workshop to now? How would you use an app to be more active and do exercise?
How do you feel about using an augmented reality app to support you to be more active?	Why would you use one? Why would you not?
Which part of the app do you feel would help you the most in doing more exercise?	Which features did you enjoy the most? Which features do you think you wouldn’t need?
Exercise selection and prescription	
Which exercises do you remember from the app?	—^a^
What did you think about the exercises that were selected for you?	Did you find the exercises easy or difficult? Have you done any of these exercises before?
What do you think children who are your age (or younger or older) would think about these exercises?	—
Augmented reality avatar experiences	
What did you think about the avatar?	What did you like about the avatar? What kind of avatar would you prefer?
How did you find the avatar’s demonstrations?	Were you able to follow the reps and rounds? Was this made clear enough on the screen?
Were there any problems with the avatar whilst you were using the app?	Did the avatar appear properly on the screen? Was the avatar the wrong size? Were there any problems with the avatar’s demonstrations?
App usability	
How do you feel about the phone we gave you for using the app?	Did you find it easy or difficult to use? What do you think about the size and display? How did you find pressing different buttons on the phone?
How did you find using the tripod that held the phone whilst you were exercising?	—
Do you feel that the equipment you were using affected your experience in any way?	Did the phone make it easy or hard to follow the demonstrations? Was the tripod getting in the way of how well you could follow the demonstrations? What do you think about the space you used for doing the exercises?
How did you find answering the questionnaire questions on the phone?	What do you think about the amount of questions? What do you think about how the questions were worded? What do you think about answering questions on an app, compared to, for example, a piece of paper?
Suggestions for improvement	
How do you think the app could be improved?	What do you think would make the avatar more useful? What do you feel we should change about the exercises?
How else do you think we could help you be more active and do more exercise?	What other technologies or equipment would you find useful?

a Not applicable.

Workshops were conducted by KS (PhD, postdoctoral research assistant, female) and HM (MSc [Res], postgraduate research assistant, female), and SW (PhD, postdoctoral researcher and senior lecturer, male, qualitative research and technology expert), and focus groups were conducted by KS and HM. The research team had previous experience in conducting mixed method and qualitative research and specific training as part of their academic qualifications. Focus groups were audio-recorded and transcribed.

### Data Analysis

Edited transcriptions were printed and reviewed in paper format, with 3 researchers (KS, SW, and HM) collaboratively conducting a coding process. Analysis combined deductive and inductive approaches [34-36]. The deductive component applied a pre-established framework based on key topics—hardware user experience, app interface, app design, AR functionality, avatar design, and exercise prescription. User experiences and suggested improvements were considered separately. Any remaining data from the discussion were inductively coded through intercoder conversations [32].

**Figure 3.**
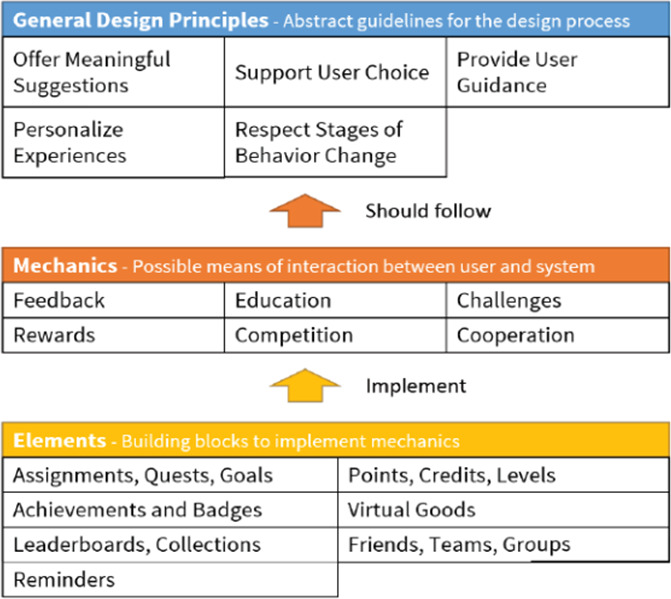
Taxonomy of motivational affordances (reproduced from Weiser et al [24], which is published under Creative Commons Attribution-NonCommercial 4.0 International [CC BY-NC 4.0 37]).

Features mentioned as suggested improvements were mapped onto the mechanisms and design elements outlined in the taxonomy of motivational affordances (Figure 3) [24]. Applying the taxonomy ensured relevant features were mapped onto evidence-based strategies and a unified terminology was used, which will aid the development and evaluation of future tools.

Moreover, 2 coders (HM and KS) independently mapped the features to the taxonomy to enhance the validity of the coding process. Subsequently, “critical friend” discussions were held to explore the suitability of the respective selections and to encourage reflexivity and enhance the dependability and credibility of data [38]. This approach also encouraged KS and HM to reflect upon their coding and provided room for considering alternative interpretations [38].

## Results

### Overview

In total, 19 primary school pupils, 28 secondary school pupils, and 3 undergraduate (UG) university students took part in the practical workshops, with 39 providing consent to take part in the subsequent gender-mixed focus groups. These were conducted in the respective educational facilities (eg, school classroom). There were 8 focus groups in total—3 with primary school students, 4 with secondary school students, and 1 with university students. Workshops and focus groups were conducted between June and December 2021 at one primary school in Gloucestershire, one secondary school in Oxfordshire, and one university in Oxfordshire (United Kingdom). Participant ages and year groups are presented in Table 2.

Participants were able to test the app for 20‐30 minutes before taking part in semistructured focus groups, which lasted between 16 and 28 minutes.

**Table 2.** Demographic characteristics of focus group participants.

Stages of education	Total participants, N	Sex, n
Primary school (year group 4-6, ages 8-11 y)	8	4 male, 4 female
Secondary school (year group 7-9, ages 12-14 y)	19	4 male, 15 female
Sixth form of secondary school (year group 12, ages 16-17 y)	9	6 male, 3 female
Undergraduate university students (ages 18-21 y)	3	2 male, 1 female
Total	39	16 male, 23 female

### User Experience

#### Overview

Overall, most participants reported that they enjoyed using the app and described it as “fun” (Y9, UG). One participant explained they enjoyed using an app as it “was fun to do exercise that you don’t usually do” (Y9). They also mentioned they would likely use it if it were available and suitable for their needs. Moreover, they felt the app could be a useful tool for promoting physical activity more broadly, and most agreed that it would help users engage more specifically with exercise. In particular, participants pointed out that the app “could build confidence” and “basic strength” (UG) to enable users to subsequently engage in more comprehensive physical activity provisions (such as group exercise classes). When comparing face-to-face exercise provisions, a participant shared that

*sometimes it’s nice to not have a scheduled time to go and see someone to do it in person. [...] If you feel insecure or feel weak, you can literally just set up the phone and do it alone [...]. It feels a lot easier.*

[UG]

When asked about long-term use, some participants mentioned they would likely use it for a long time, whereas others stated they would likely use it for the shorter term only, with one describing they would engage with it for “two weeks and then get bored with it” (Y8). In contrast, other participants highlighted that the app could be helpful as a “prompt” to exercise, and it is “easy to follow and to pick up and make a habit of” (UG).

#### AR Functionality

During the focus groups, participants specifically discussed their experiences of using the app with regard to the AR functionality, app design, onboarding processes, accessibility, and safety. In addition to the iPhones (model 12, screen size 6.06 inches; Apple Inc), participants in this study were given a tripod to ensure they could execute the exercises without holding the phone. Participants generally found it useful to have a digital exercise demonstration, compared with paper-based drawings or text. Participants shared that using the AR app makes accessing exercise “quick” and “easy” (UG). Participants shared that the AR avatar felt like a “training partner” who provided a bit more motivation and “[made] the exercise more fun” and “might make […] someone feel like one of their friends is doing the same” (UG). Furthermore, participants felt that for clinical users who may wish to do activities without face-to-face supervision, the AR functionality could be useful to provide demonstrations and maintain a sense of personalization and care. Participants liked being able to watch the demonstration for as long as they needed to and “didn’t feel rushed” (UG), as there was no specific time allocation per exercise. Participants agreed that having a virtual exercise demonstration is useful, especially if users had insufficient knowledge to execute specific exercises.

In comparison, there was no consensus among participants with regard to the use of AR compared with more traditional (2D) video demonstrations. One participant mentioned that “there are more problems presented by the AR than solved by the AR” (Y12), hinting at technical difficulties that occurred when scanning the room to allow the avatar to appear. Participants mentioned that they had to scan the surroundings with the phone for the AR avatar to appear before each exercise, which some stated was “frustrating” (Y8, UG), and others felt like they should not have to physically move the phone each time to make the avatar appear. It should be noted that this was not an issue for everyone, with some participants saying that “it worked quite quickly” (Y8). It also transpired that at times, the avatar would appear in a different location to the one scanned, or appear too large or too small. Moreover, some participants mentioned that it was difficult to follow exercises that required them to be seated or lying down on the floor, as they could no longer see the phone screen. It was suggested that using videos would allow users to move the phone without impacting the exercise demonstration. On the other hand, some participants stated that the use of an avatar was unique to this app and generated a more enjoyable experience compared with traditional apps (ie, those using videos), as it made the exercises feel more like a game. One participant said that,

*[...] it was really fun and enjoyable because while I was doing it, I didn’t really notice I was doing exercise.*

[Y5/6]

#### App Design

Most participants stated that they merely focused on the avatar, without reading the other information presented on the screen, whereas some read the text before looking at the avatar. Among users who indicated they read the instructions, some found it difficult to decipher whether they were asked to perform 1 round, 1 set, or multiple sets. It was evident that text presented in bold was perceived as important and was prioritized by some participants before beginning the exercise.

#### Onboarding Process

Some participants questioned the relevance of some of the questions, for example, with regard to the ability to keep their balance on a slippery surface, and also mentioned that some of the questions felt repetitive. Others appreciated that the individual questions were needed for the subsequent exercise prescription, and older users mentioned they liked that there would be scope for progression.

Participants stated that an option to skip a question they felt uncomfortable answering would be beneficial as “someone might be worried that if [...] they find it hard to do something, they might not like people knowing that” (Y5/6). Participants appreciated that the questionnaires did not ask them for any input on their height and weight, as they perceived this may be potentially uncomfortable for some users. Moreover, questions that enabled more tailored exercises were welcomed as they would “feel more motivated because it’s actually targeted [...] personally, instead of just a generic app” (UG).

Regarding the number of questions, there was no consensus among participants. Some felt the questionnaires were too long, and they would potentially skip questions, respond randomly, or cease use of the app. Others stated the questionnaires were “quite short” (Y4) and not “too long or bothersome” (UG). One participant appreciated that “[...] for the people who actually need it, [they] would pay attention to what is actually going on because they want to get better” (Y9), hinting that intended users (ie, children with cancer) would also be more likely to appreciate the need for onboarding questions.

It was also discussed that most felt the questions were easy to read; however, others stated they might need to be shortened for younger users. One participant (Y5/6) mentioned that the questions should be as specific as possible, as they struggled to select an answer when the question was not clear (eg, asking about their ability to carry a heavy book, with confusion on what “heavy” would entail).

In regard to the answer options, most participants felt that 5 options were sufficient, and the wording should be clearer to effectively distinguish between options (eg, “sometimes” vs “almost never”).

#### Exercise Prescription

Most participants stated that the prescribed exercise intensity was achievable, although a few participants reported that some of the exercises were too easy. The selected exercises were deemed to be suitable, with a “good range” and appropriate provision of exercises done in a lying, sitting, and standing position. One participant felt this made it “interesting” and “fun” (Y9).

Participants felt that a gradual intensity progression is helpful in regard to a decrease or increase in intensity or repetitions based on previous performance or feedback input from users. The rate of perceived exertion scale (Figure 2) was well-received, and participants found it easy to select the relevant score.

#### Accessibility

Some participants stated that they found it beneficial not to need specific equipment (ie, other than a chair), whereas others mentioned they would prefer exercise sessions that require no equipment at all. One participant added that the app was also beneficial for those with limited access to appropriate outdoor space for exercising as “you don’t need a big field to do exercise, so even if you live in a flat […] you can still do it” (Y5/6). It was also recognized that paying for exercise apps, such as this one, could be a barrier for some users, as expressed by one, “it’s really good to have it free” (Y7).

#### Safety

A couple of participants also discussed their experiences with data and usage safety, including concerns regarding how much, and what type of information was collected, as well as how this would be shared. One participant hinted that the length of the onboarding questionnaires may “[make them] a bit suspicious of what is happening so [would] delete it” (Y9). One participant mentioned their parents may share these concerns and would encourage them not to provide personal information, such as their real age.

#### Avatar Design

Participants generally agreed that the avatar design was appropriate for a variety of ages, with no suggestions made on specific features that should be changed as a default. Participants commented that the avatar was “cute and funny” (Y9), and the design allowed for easy interpretation of the exercise demonstrations. Older participants highlighted that they appreciated the semirealistic design and felt that the avatar’s body shape was helpful not to “feel pressure of ‘you have to look like this’” (UG) but rather encouraged them to focus on being physically active for enjoyment.

### Suggested Improvements

Throughout the focus groups, participants voiced suggestions for future improvements of the app. Table 3 contains an overview of suggested improvements linked to the following motivational affordance mechanisms: user education, challenges, feedback, cooperation, and comparison.

**Table 3.** Summary of suggested improvements by motivational affordance mechanisms.

Category	Participant quote	Recommendation	Motivational affordance design elements
User education			
Exercise instructions	“I think if it explains to you what it does or [help with], […] that would be more educational” (UG)^a^ “[...] I didn’t actually know how long I was doing it for.” (Y4) ^b^“[...] It didn’t [...] say per leg or all together.” (Y12)^g^ “[...] It could say [...] you need a chair [...].” (Y12) “[…] I would prefer more text or description of what we’re going to do” (UG) “I think it would definitely help especially when you are doing an exercise when you can’t […] directly see the screen, the audio telling you the next exercise is about to start would definitely help someone notice when to move onto the next one” (Y12)	Provide detailed (written or audio) instructions explaining purpose, repetitions, and equipment	Assignments
Visual demonstration aids	“It would be better if it had arrows of [...] where to move.” (Y9)^f^	Implement additional visual guidance to support movement execution	Assignments
Duration	“[...] there [could] be a timer in front of you so you could see [...] how long there is to do this exercise.” (Y12) “[...] There should maybe be an option to do a countdown [...].” (Y7)^d^	Consider use of countdowns or timers	Assignments and goals
Order	“I would try [to] space out the chair exercises [...]. [...] So maybe do a sitting down one, a lying down [one] and then a standing up [one].” (Y5/6)^c^	Ensure sitting, lying, and standing exercises are included and interspersed	Assignments
Exercise type and variety	“I guess it would be good to have more variety [...]” (Y12) “[...] There should be options on what you want to work on, like if you want to work on your legs [...], on your arms, or you want to do your whole body.” (Y9)	Give a variety of exercises and allow user choice for type of exercises (including strength and mobility)	Assignments, quests, and goals
Rest	“I think [...] there should be a rest time.” (Y9) “[...] If it was as long as you like, then I would give up. I would lay down and forget about it.” (Y9)	Implement specific durations for rest	Assignments
Equipment options	“[...] You could do an option so people that aren’t used to exercising [...] can have a chair with them [...].” (Y5/6)	Provide exercises using equipment to facilitate safe and appropriate exercise performance	Assignments and quests
Challenges			
Setup	“[...] There should be [...] a few different levels, so an easy, a medium and a hard [...].” (Y4) “I think it would be nice [...] before you go to the exercise you can type in how much you want to do [...].” (Y12)	Provide different levels of intensity to account for previous experience and current capabilities and allow users to select their own	Levels, quests, and goals
Intensity progression	“[...] It [should be] updated [...] every two weeks [...]. The exercise levels could change, [...] get harder.” (Y8)^e^ “[…] If you found it too hard, it can make the exercises a bit easier next time” (Y9) “[...] Whenever you finish an activity, you can write down what was easy or hard for you [...].” (Y7)	Allow intensity progression or regression and user input to gain feedback on perceived exercise intensity	Assignments, quests, goals, and levels
Progress overview	“A form of motivation would be a progress bar, [...] and over time you can see [how] well you improve.” (Y12)	Provide progress overview graphs, bars, or charts as motivational tool	Points, achievements, and badges
Feedback			
Compliance	“[…] There could be a sensor, it could make sure, seeing what you are doing, like saying out of 10, it knows what you are doing” (Y5/6)	Consider sensor technology to track movements and give feedback on execution	Points and achievements
Encouragement	“I thought it was a bit quiet, so “you can do this,” or like “you’ve done great” or something” (Y9)	Provide audio encouragement during or between exercises	Achievements
Cooperation and comparison			
Group exercise	“Maybe you could choose how many people do the workout, so if you want to do it as a family then you could say partner round or how many people are playing with and then you could have three different avatars […]” (Y5/6) “It would be a good idea if, […] you could FaceTime a friend whilst you’re doing the exercise or you could have like a split screen thing where you both do it and you can see what each other are doing and like talk to them whilst doing it” (Y7)	Give options for group exercise with invited users	Friends, teams, and groups
Social support	“Because your friends would probably encourage you if you say this is hard, they would encourage you” (Y7)	Allow users to interact with others via call or chat feature	Friends, teams, and groups
Performance comparison	“Like a scoreboard with other people’s scores on it.” (Y5/6)	Allow comparison between users via visual aids	Leaderboards

a Undergraduate (UG) focus group.

b Y4: Year 4 focus group.

c Y5/6: Combined Year 5/6 focus group.

d Y7: Year 7 focus group.

e Y8: Year 8 focus group.

f Y9: Year 9 focus group.

g Y12: Year 12 focus group.

In addition to these improvements, participants discussed options for incorporating gamification within the app, which was mentioned to hold potential to attract users who usually do not enjoy exercise. One participant added that “[...] rather than playing games [...], [they] would be doing exercise” (Y7). They felt it could be useful to use young people’s interest in computer gaming or other activities and amalgamate this with the exercise-related features of an app. Table 4 contains an overview of gamified features proposed by participants, linked to challenges and rewards as per the motivational affordance mechanisms.

**Table 4.** Overview of suggested improvements for gamification by motivational affordance mechanisms.

Category	Participant quote	Recommendation	Motivational affordance design elements
Challenges			
Exercise tasks	“[...] If you are jumping on the spot, [...] you can try and beat your high score [...].” (Y4)^a^	Give personalized activity tasks	Quests and challenges
Cognition	“[...] You can add games that could teach them about Maths and English [...].” (Y7)^b^ “You could [...] match the [...] name of the exercise to the actual exercise.” (Y8)^c^	Integrate games related to school subjects or logical thinking	Quests
Special interest	“[...] It might be good to teach them activities, [like] how to shoot a basketball, how to dribble a football.” (Y7) “[...] There could be [...] vocal exercises for people who like singing.” (Y7)	Incorporate activities to train special interests	Quests
Virtual world	“[...] You could make the avatar do games, like football or basketball [...].” (Y4) “[...] The avatar could do stuff at home, like as if it’s them but in the game, [...] like going to school and things [...].” (Y7)	Create virtual world for the avatar to complete tasks	Quests
Rewards			
Virtual rewards	“[...] If you reach your [...] target for one day of exercise you then [...] unlock the points and then you can get something to customize your avatar a bit more [...].” (Y9)^d^ “[...] At the start of the app you could have the yellow one and blue one but then when you complete [...] 10 exercises, you can unlock other [...] [colors].” (Y9) “[...] If you do [the exercise] amazingly, you could get [...] stars and you could trade it in for [...] a little fun game play.” (Y5/6)^e^	Provide rewards (eg, via avatar customization) for exercise achievements	Credits, achievements, and badges

a Y4: Year 4 pupils focus group.

b Y7: Year 7 focus group.

c Y8: Year 8 focus group.

d Y9: Year 9 focus group.

e Y5/6: Combined Year 5/6 pupils focus group.

In addition to the suggested improvements shown in Tables3  4, and outside of the scope of features related to motivational affordances, participants highlighted that equipment for using the app should be user-friendly. For example, phone screens should be of sufficient size (>6 inches), and tripods should be sturdy and easy to adjust. Moreover, customization of the app interface (eg, by giving options to change background colors), or of the avatar (eg, by allowing users to change its features, such as age, gender, skin color, hair, body composition, and clothes, or giving nonhuman options, such as, animals or fantasy characters) would be useful and could enhance engagement with the exercise. It was also suggested that allowing users to play their own music or select from a choice of songs could be motivating.

## Discussion

### Principal Findings

This study explored user experiences of an AR exercise app and mapped users’ suggested improvements against motivational affordance mechanisms and design elements [24]. Most participants reported that they enjoyed using the app. Participants found the demonstrations and varied exercises useful but expressed some concerns regarding data safety and functionality of the AR avatar. Suggestions included improvements, such as additional educational components, incorporating challenges and rewards, as well as a customizable avatar and a social support feature, such as the possibility of exercising with family or friends. Additionally, some suggestions, such as the integration of audio instructions for a greater inclusive design, were made. Finally, users also expressed concerns about personal data safety. When mapped against the motivational affordances taxonomy, the suggested improvements aligned with mechanisms of user education, challenges, feedback, cooperation, and comparison. This study underlines the importance of incorporating a variety of motivational affordances to account for different user needs and preferences.

### Motivational Affordances

Within this study, participants’ suggestions frequently mapped against the taxonomy of motivational affordances [24]. It is promising that motivational affordances incorporated within the current app, such as user education on how to perform exercise, seem to be particularly effective in improving physical activity levels among patients with cancer in other research [39]. Participants in our study also suggested new features, such as audio instructions, visual progress bars, and the ability to connect with other users, highlighting the need to further develop design features, such as achievements, credits, and friends [24].

While current studies may assess the general usefulness of mobile apps, a deeper exploration is needed to determine how specific features drive engagement and thus encourage long-term physical activity, especially in populations, such as pediatric oncology, which we know face additional barriers to exercise. Currently, we may only understand the usefulness of motivational affordances, and further research should delve deeper into the mechanisms through which the features work, for example, via belief in capabilities and intentions [40]. Investigating these underlying mechanisms could help optimize app design and ensure that interventions are effectively promoting behavior change, and not only short-term app use. Long-term engagement is particularly important for survivors of children with cancer as physical activity levels are often lower than noncancer controls [41], and observational studies have identified associations between sedentary behavior and adverse effects on cardiometabolic and bone health [42].

### Gamification

For end users to use and positively benefit from an app, they must actively engage with it. Our findings indicated that incorporating challenges and rewards is preferred, which is in line with other app research [43]. However, it has to be considered that when using the app within clinical populations, there may be contraindications (eg, treatment-related side effects) that prohibit users from engaging with the app, or some of its features, for a period of time. When programming the app to provide challenges and rewards, such as continuous usage streaks or increases in exercise prescriptions, it is crucial to also incorporate rewards for the performance of tasks achievable by all users. Additionally, if participation is affected by a reason outside of the users’ control (eg, treatment), they should not be negatively affected (eg, losing a daily streak or points). The approach of personalized gamification has been highlighted by Mahmoudi et al [44], with the addition of customization, where users can select elements they wish to use. Customization of the app interface and avatar was a frequent suggestion within this study. Avatar-based interventions, and specifically customizable avatars, have been shown to promote health-related behavior change, including exercise [45]. Within the motivational affordance taxonomy, there is no clearly defined mechanism dedicated to “customization” [24]. While customization as a reward or as a transactional element involving earned coins may be conceptually aligned with the motivational affordances design features of credits or achievements, there is no explicit mechanism pointing toward users customizing features (eg, interface or avatar) without first fulfilling certain criteria or earning that capability. While customization may be a popular demand, future research should explore whether being able to personalize apps is motivating in itself or whether the motivation is attached to the act of “earning” the reward in order to do so.

### Social Support

The results showed that having a social support feature, such as being able to exercise with family members or friends, may be beneficial for app engagement and links well to friends, teams, and group mechanisms within the taxonomy of motivational affordances [24].

Family apps have been seen to have promising effects on physical activity levels and psychosocial outcomes by promoting child-parent exercise [46]. Including social features in exercise apps for children with cancer may enhance engagement and motivation. One study [47] found that physical and social activities merged when ambassadors (ie, peers or siblings) participated alongside survivors of childhood cancer, supporting enjoyment and adherence. Integrating peer interactions into digital platforms could replicate these benefits, promoting physical activity and well-being. While the current app does not have this feature due to the restrictions of data protection and safety, this concept may be of interest when considering the isolation and reduced opportunities for social interaction often experienced by children with cancer [48].

Interestingly, 1 participant revealed a different perspective and highlighted that the app could be a useful tool for those who do not have family members or friends who exercise. It was mentioned that the AR avatar could serve as a form of “digital friend” who could positively influence exercise participation, which was in line with findings from a study conducted by Thorsteinsson et al [47]. There is some initial research on a sense of belonging and digital support received from apps that can positively influence physical activity levels [49]; however, the findings of this study underline the importance of exploring this concept more comprehensively.

### Accessibility

The results highlight a need to enhance accessibility within mHealth applications, particularly through the extension of existing motivational affordance mechanisms, such as assignments, to better accommodate diverse user needs and promote inclusive design. For instance, the inclusion of a text-to-speech function was recommended, which would provide meaningful support for users with reading difficulties or visual impairments. This recommendation aligns with existing research emphasizing the importance of designing inclusive digital experiences [50]. The evidence highlights the importance of accessibility features, such as voice control, voice over, color contrast, and easy access controls, to ensure apps are usable for individuals with disabilities [50]. These features are particularly relevant in physical activity apps, where ease of use and clear instructions are essential for safe engagement. For example, individuals with visual impairments may rely on voice commands and audio feedback to navigate exercise programs, while those with motor disabilities may benefit from customizable controls that allow for seamless interaction. Therefore, app developers should consider the inclusion of such features to ensure inclusivity and enhance user satisfaction across diverse populations.

### Data Safety

Results highlight concerns regarding the appropriateness of the app collecting and potentially sharing personal data (eg, age). It is important to recognize that the use of an app that requires any user input should be accompanied by relevant training and information for users. It is expected that, by providing extensive information to users and their parents or guardians, concerns may be mitigated effectively, with users satisfied and subsequently willing to provide accurate information. It has been widely acknowledged that the increased volume of data available through the use of mHealth tools, such as apps, can indeed be used to provide better services if current data protection and sharing regulations are adhered to [22]. Looking ahead, due to the frequent multicomponent treatment approach for children and young people with cancer, it would be crucial to explore how user data within apps (eg, rate of perceived exertion and exercise duration and intensity) can be integrated within digital health records for a more comprehensive treatment approach. As part of such evaluations, specific mHealth reporting and assessment guidelines, such as the mHealth Evidence Reporting and Assessment checklist, should be used [51].

### Strengths and Limitations

This study provides a rich and in-depth understanding of user experiences when using the app, allowing researchers to study potential unexpected patterns in how users interact with the different app features. Mapping results with motivational affordances allows for a better understanding of certain features that might be more motivating and support app engagement within this population. Our findings can inform the development of effective mHealth tools that support behavior change in the field of exercise and physical activity as well as guide future interventions, including more personalized, user-centered approaches to digital health technology.

Despite our study’s strengths, it is important to acknowledge limitations. First, this study recruited healthy participants. While some perceptions of the app’s functionality are expected to be comparable between patients with cancer and healthy age-matched peers, there may be differences in the usability of the app between these 2 populations. Some pediatric patients with cancer may have a unique motivation with regards to exercise and physical activity, as well as different physical and cognitive needs and preferences; user experiences of some features could differ. Currently, the AR app is at technology readiness level 5. Future studies with the intended clinical population would progress the app to technology readiness level 6.

Second, the study targeted a broad age range (8‐21 y), while the app itself remained largely static across all ages. While differences in cognitive ability, physical capability, accessibility, and design preferences may mean that some features are less engaging for certain age groups, given the lack of existing AR apps for this population. This approach is also pragmatic, considering the high costs associated with technology development, allowing initial feedback to inform improvements before investing in age-specific customizations.

Third, this study focused on short-term app interaction. Given that the app is designed to support exercise prescriptions over several weeks, the brief duration prevented the assessment of features influencing longer-term adherence. Although the controlled setting allowed for the collection of immediate feedback, it did not capture the evolving experiences users may have during extended use. Future studies should incorporate longitudinal testing to better understand long-term engagement and identify which features support ongoing use.

Finally, there was also the potential of researcher bias in the study, as the investigators had a background in sport and exercise science, which may have influenced how data were interpreted or which themes were prioritized.

Despite these limitations, the study provides valuable foundational evidence to guide improvements to the app and inform future research on implementing mHealth tools within pediatric exercise interventions.

### Conclusion

Theory-driven evidence for best practice on mHealth apps is often inconsistent or not focused on the overall user experience. This study explored young people’s experiences of an AR exercise app and mapped suggested improvements onto the motivational affordance mechanisms and design elements. Users identified several ways the app could be strengthened, including the addition of educational content, challenges and rewards, a customizable avatar, and social features that enable exercising with family or friends. Suggestions also highlighted the need for greater inclusivity through features, such as audio instructions, and clear safeguards around the security of personal data. Mapping these recommendations onto the motivational affordance taxonomy revealed alignment with mechanisms, such as assignments, achievements, friends, groups, and credits, underscoring the diversity of motivational pathways that can support engagement. Further research directly involving children and young people with a childhood cancer diagnosis is planned to further explore the preferences of those facing unique barriers to physical activity and exercise. Such work will also be crucial for advancing the technology readiness level of the intervention by testing the app with the intended population in real-world settings.

## Supplementary material

10.2196/76855 Checklist 1 COREQ checklist.

## Abbreviations

AR: augmented reality
COREQ: Consolidated Criteria for Reporting Qualitative Research
mHealth: mobile health
UG: undergraduate
